# Gambling Habits and Attitudes among Athlete and Non-Athlete High School Students in Skåne Region, Sweden

**DOI:** 10.1007/s10899-024-10333-3

**Published:** 2024-07-12

**Authors:** Molly Miles, Mitchell Andersson, Emma Claesdotter-Knutsson, Sabina Kapetanovic, Anders Håkansson

**Affiliations:** 1 Faculty of Medicine, Department of Clinical Sciences Lund, Lund University, Baravägen 1, Psychiatry, Lund, S-221 00 Sweden; 2 Malmö Addiction Center, Region Skåne, Sweden; 3 Department of Child and Adolescent Psychiatry’, Region Skåne, Sweden; 4 Högskolan Väst, Western University, Trollhättan, Sweden; 5 Stockholm University, Stockholm, Sweden

**Keywords:** Gambling disorder, Problem gambling, Elite athlete, Adolescent, Sports psychology, High school athletes

## Abstract

Previous literature has reported increased rates of gambling problems in athletes compared to non-athletes. More liberal gambling-related attitudes have been suggested as a reason, although this rarely has been researched. The present study aimed to examine gambling experience, gambling problems, and gambling-related attitudes and parental gambling experience in high school students, comparing student-athletes to students at conventional schools. This is a cross-sectional web survey study in high school students (*N* = 473, 53% at sports high schools, 57% male) at eleven schools in the Skåne region, Sweden, who answered a web survey addressed gambling experiences, parental gambling and gambling-related attitudes, and included validated screening instruments for gambling problems and psychological distress. A history of any gambling was common and increased with age. Problem gambling was detected in 10% (13% of males and 5% of females, *p* < .001), and was associated with paternal and maternal gambling but not with psychological distress. Sports high school students were not more likely (9%) than other students (10%) to endorse gambling problems and history of each gambling type. However, paternal (but not maternal) gambling was more commonly reported in athletes, who also had more positive attitudes to gambling’s effects on society and gambling availability. In contrast to other studies, this study did not demonstrate higher prevalence of gambling or gambling problems among young athletes than among other students, but liberal attitudes towards gambling, and experience of parental gambling on the father’s side, were more common among athletes than among non-athletes. Gambling attitudes in adolescents may need to be targeted in future preventive efforts in young athletes and others.

**Supplementary Information:**

The online version contains supplementary material available at 10.1007/s10899-024-10333-3.

## Background

Gambling disorder (GD) is expressed with a consistent and frequent behavior of gambling associated with detrimental effects on an individual’s mental health and financial situation. The fifth edition of the Diagnostic and Statistical Manual (DSM-5, American Psychiatric Association, 2013) defines GD as a criteria-based diagnosis and an addictive disorder (Potenza et al., 2019). GD often leads to profound financial problems for the individual, accompanied by severe psychiatric complications and heightened risk for engaging in criminal behavior (Langham et al., 2016; Moghaddam et al., 2015). The lifetime prevalence of problem gambling has been reported to range from around 1% to almost 6%, varying because of factors like different study settings and diagnostic criteria (Calado & Griffiths, 2016).

The prevalence of GD has been shown to differ between the sexes; the ratio is at least 2:1 in males compared to females. Well-known risk factors of GD include mental health comorbidity, and socio-economic and psycho-social problems (Potenza et al., 2019). In Sweden, the setting studied here, the age group 16–29 years has the highest incidence of problem gambling, where around 5% have a risk behavior according to a survey using the Problem Gambling Severity Index (PGSI, Abbott et al., 2018). Approximately 34% of upper secondary school students in grade two reported that they had gambled at least once during the past 12 months, and 5% met the criteria for problem gambling (Public Health Agency of Sweden, 2015).

Previous studies have shown that both athletes who are active within Swedish sports in general (Håkansson et al., 2018), and specifically team sports (Håkansson et al., 2020), have a higher risk of developing problem gambling. In a cross-sectional online survey conducted by Vinberg and co-workers, findings unveiled a higher prevalence of problem gambling among male elite athletes (engaged in ice hockey, football, floorball, and basketball), ranging from 11 to 14%, compared to a male sample aged 16–39 from general population (4%) (Vinberg et al., 2020). Various facets of sports activities often intersect with the realm of gambling. This includes betting, sponsorship by betting companies, promotion of gambling related to sports (Deans et al., 2017), and a risk of match fixing, i.e., the exposure of gambling fraud (Moriconi & de Cima, 2020). This may make athletes and others involved in sports more vulnerable to develop issues with gambling (Vinberg et al., 2021). Altogether, it has been hypothesized that attitudes towards gambling, such as being in an environment that is liberal with respect to gambling habits, may increase the risk of problem gambling in athletes (Vinberg et al., 2021). Likewise, permissive attitudes may derive from parents; parental gambling habits have been suspected to increase the risk of adolescent gambling (Vachon et al., 2004), and gambling problems in youth have been linked to the reporting of gambling with adults (Rahman et al., 2014). Thus, the examination of attitudes and experiences of gambling with parents may be of importance in the assessment of adolescent gambling.

The existing knowledge and data on the subject are confined to certain sports, necessitating further studies to grasp the extent of the phenomenon. In addition, few studies have assessed gambling patterns in younger individuals enrolled in high school (Weiss, 2010), and compared them to a similar non-athlete sample from the general population.

The aim of this study was to describe differences in gambling behavior and attitudes towards gambling in upper secondary school students in Skåne region, Sweden. In addition, we investigated whether problem gambling has a higher prevalence in young athletes compared to non-athletes and adults. Specifically, the study aimed to answer the following research questions: How common is problem gambling in high school students in athletes and non-athletes, in females and males, in individuals with or without other psychiatric comorbidities, and, among student-athletes, between individuals engaged in individual and team sports, respectively? How common is it to have gambled for money per each gambling type in the different groups listed above? Do attitudes towards gambling, and parental gambling, differ between individuals attending a sports class vs. non-sports class? In individuals identified as problem gamblers, how do the responses to the questions concerning attitudes toward gambling and psychological distress distribute?

## Methods

The present study is a web survey addressing upper secondary school students (year 10–13) in the Skåne region in southern Sweden.

### Setting

As in many other European countries, the legal age of gambling for money in Sweden is 18 years. The age limit applies to all types of gambling, although the exception is the three state-owned land-based casinos owned by a subdivision of the state-owned operator (previously a monopoly), AB Svenska Spel, where the age limit is 20 years. Eighteen is also the official age when one is considered an adult in Sweden. Today, the Swedish gambling market consists of around 80 licensed operators. The operators are individually responsible for verifying the age of their gamblers. The Swedish Gambling Authority works actively to control that the operators comply with the age limits. If age limits are not respected, the operator risks losing its license. There are also regulations concerning marketing of gambling, which forbids gambling operators from aiming their marketing strategies at children and youth under the age of 18 (Swedish Gambling Authority, 2018).

### Study Procedure

The recruitment process started with contacting principals of 20 upper secondary schools in the Skåne region, Sweden, one of them representing several schools in one area. Contact was both conducted through phone calls and emails with written information. The principals were given information regarding the web survey and were asked to allow the school to participate. Out of those 20, eleven principals accepted the invitation. After consent was obtained, the survey was either distributed through the principal to each of the teachers within the school, or directly to specific teachers. A link to the web survey, written information and a consent form were then sent to the teachers. The web survey was presented to the students during mentoring time when they got designated time to fill out the survey.

The current web survey was conducted from January 16 to February 17, 2023, and was closed when 472 complete responses were received. A reminder was sent out to the schools on January 30. Participation was completely voluntary, and all answers were anonymized. Participants were required to provide informed consent to gain access to the survey. In total, 682 individuals entered the survey, 661 provided informed consent and agreed to participate in the survey, and 472 completed the survey in its entirety.

### Measures

The National Opinion Research Center DSM-IV Screen for Gambling-CLiP (NODS-CLiP) was used for the assessment of lifetime problem gambling. It consists of three questions to detect the risk of gambling problems. Answering “yes” to any of the questions gave 1 point, with a maximum total of three points. Zero points was regarded as no risk gambling, while 1–3 points indicated a risk of gambling problems, and that further assessment of gambling problems should be conducted (Toce-Gerstein et al., 2009).

In the beginning of the survey, participants were asked about whether they attended a sports high school/sports class or not, and if yes, which specific sport the participant’s curriculum involved. Sports classes/schools in this study were any of the three categories of sports-oriented high school educations present in the Swedish system, i.e. classes which have a clear sports orientation, with either a local or national application procedure. Thereby, for these high school students, their high school curriculum has involved a substantial involvement in their specific sport during school hours. Additional information extracted in the present study included age, gender, living conditions, grade/year in school, and whether the participant has been diagnosed with attention deficit hyperactivity disorder (ADHD), attention deficit disorder (ADD), autism spectrum disorder (ASD), or a learning disability diagnosis such as dyslexia (data not shown). Gender referred to the gender that the participant identified as, and participants were divided into males and “non-males” in the analysis, where the later included females, other gender identity and those who preferred not to answer the question. The latter categorization was decided based on the assumption that males would have a higher prevalence of problem gambling (Calado & Griffiths, 2016; Abbott et al., 2018).

More specific questions concerning gambling behavior were asked, such as if the participant ever had gambled for money and if so, what type of gambling. Eight gambling types were included, among which two are land-based (land-based sports betting such as in gambling stores and similar, and physical poker gambling), and six were online-based (online casino, poker, sports betting, horse race betting, bingo and gambling within computer games). Land-based casino (available on three locations in the country as part of a state monopoly, with a 20-year age limit) and land-based horse race betting (in regulated horse race tracks) were not included and judged to be highly unlikely to be endorsed by participants, due to stricter on-site controls of age limit. The participants were also asked to answer questions regarding their attitude towards gambling-related commercials, gambling legislation and their moral position on gambling issues. The web survey also included questions about parents’ gambling habits and attitudes. Gambling habits were evaluated with questions about any gambling at any point in life and during the past 30 days for each of the different gambling types.

Lastly, we measured psychological distress of the participants using the Kessler-6 scale. The scale consists of six items measuring self-reported symptoms of depression and anxiety over the previous six months. Each item is scored between 0 and 4 points and the total score is summarized from 0 to 24 (Furukawa et al., 2003). In this study, a total score of five or more was considered to represent psychological distress on at least a moderate level. If a participant chose to not answer all the questions, but still had at least 5 points on the remaining five questions, that individual was included in the analysis as a person with psychological distress. Also, if a participant only left out their answer on one question and had a total score of 0 excluding the skipped question, that individual was also included in the analysis, as not endorsing psychological distress.

The answers of the only individual who reported 15 as age are not visible in the tables sorted by age, due to the risk of identification. One individual stated to be practicing dance as a sport, but later reported to attend a non-sports class and was therefore included in the non-athlete group. Another participant was excluded because they reported their age to be “1516”, making it impossible to determine whether the individual was referring to 15 or 16.

The survey also touched on other health issues (including history of concussions in sports) that were not addressed in this study, and which are reported elsewhere (Andersson et al., 2024).

### Statistical Methods

Statistical tests were conducted using IBM SPSS Statistics v29 and figures were created in R using *ggplot*() and *gridExtra*(). Pearson chi-squared tests (dichotomous factors) and linear-by-linear associations (age and the Kessler-6 scoring) were performed. We reported effect sizes using Phi (φ) for 2 × 2, Cramer’s V (*V*) for larger contingency table analyses, and point-biserial Pearson correlation coefficients to describe the strength and direction of linear-by-linear associations. Adjusted standardized residuals from chi-squared tests were assessed to determine where differences existed. In total, 21 cases had any missing data for psychological distress. Among them, 12 were excluded and nine could be categorized as suffering from psychological distress, because the available data summed up to a value of five or more. Due to the exploratory nature of our research questions, we did not adjust for multiple testing.

### Ethical Approval

The study was assessed by the Swedish Ethics Review Authority, which concluded that the study did not address any data from identified individuals, and therefore did not require ethics permission according to Swedish ethics legislation (file number 2020–07246).

## Results

Out of the 472 participants, 52.8% (*n* = 249) answered yes to the question about whether they attended a sports-oriented class or not. 57% (*n* = 269) of the participants were males, and 40.7% (*n* = 192) were females, 1.3% (*n* = 6) identified as “other” and 1.1% (*n* = 5) preferred not to answer the question.

### How Common is Problem Gambling in Upper Secondary School Students?

**Fig. 1:**
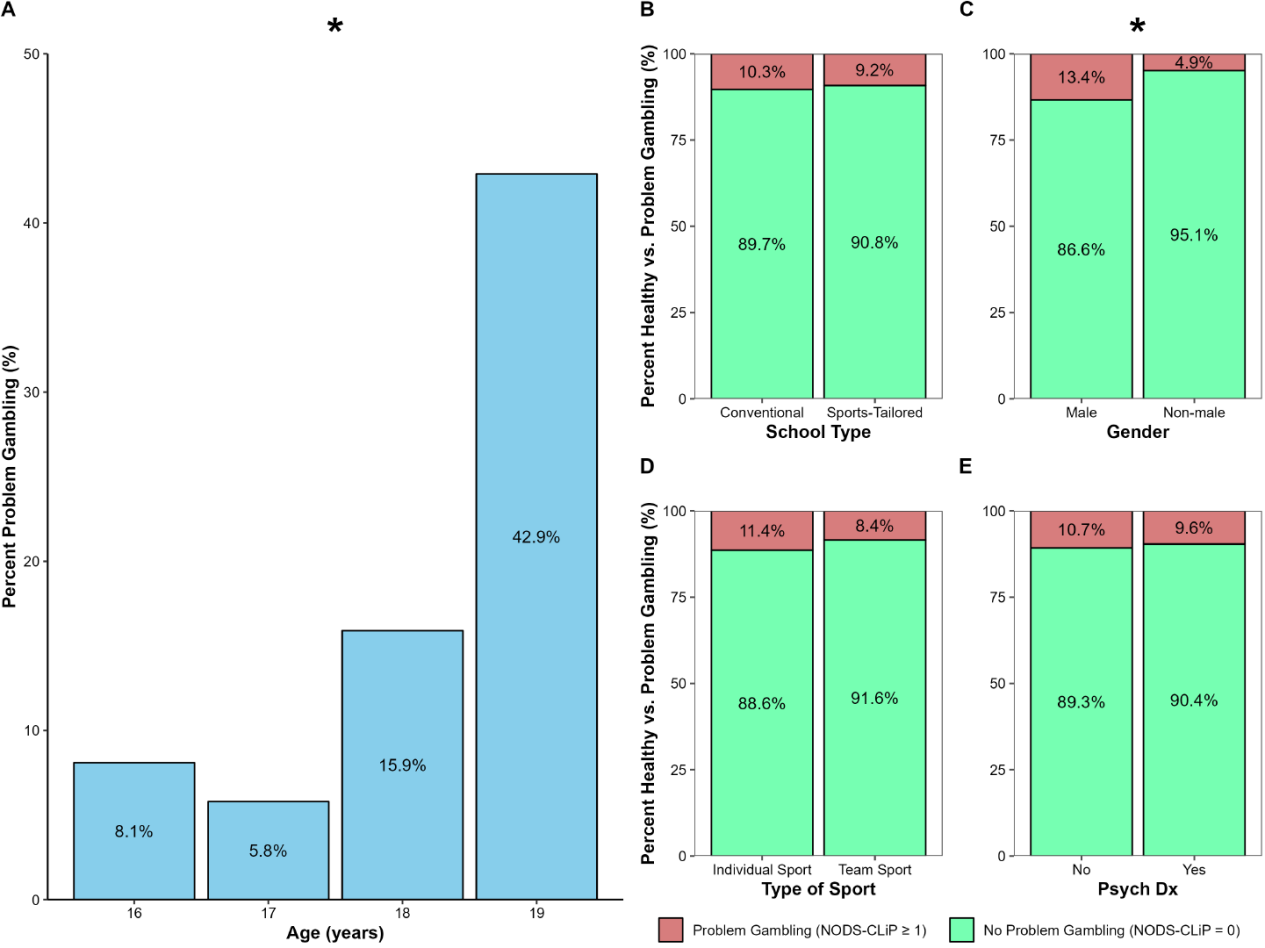
Prevalence of Problem Gambling by Demographics. Prevalence of problem gambling by age (**A**), school type (**B**), gender (**C**), type of sport (**D**), and psychiatric diagnosis (**E**). Psych Dx = Psychiatric diagnosis (i.e., attention deficit hyperactivity disorder, attention deficit disorder, autism spectrum disorder, or learning disorder). Problem gambling was associated with increasing age and gender. *p < .05

Among the 472 participants who completed the form, 9.7% (*n* = 46) responded yes to at least one of the NODS-CLiP-questions, indicating problem gambling. There was no significant difference in problem gambling between individuals attending sport classes (9.2%) compared with individuals in non-sport classes (10.3%), χ^2^(1) = 0.16, *p* = .69. Also, there was no significant difference in gambling problems between those who reported to be participating in team sports (8.4%) compared with those engaged in individual sports (11.4%), χ^2^(1) = 0.59, *p* = .46. The prevalence of problem gambling was higher in males (13.4%), compared with non-males (4.9%), χ^2^(1) = 9.41, *p* = .002, φ = 0.14. An increasing trend in problem gambling was seen with age (16–19), χ^2^(1) = 11.11, *r* = .154, *p* < .001. When comparing the psychiatric diagnoses assessed here, ADHD/ADD, autism and learning disability, there was no significant association with problem gambling, χ^2^(1) = 0.07, *p* = .80 (Fig. 1).

### How Common is Gambling by Gambling Type?

The specific gambling types included casino games online, betting on horses, sports betting in stores, sports betting online, online poker, physical cards/poker, online bingo, and computer games involving money. A total of 247 participants claimed to never have tried any of the gambling types mentioned above.

**Fig. 2:**
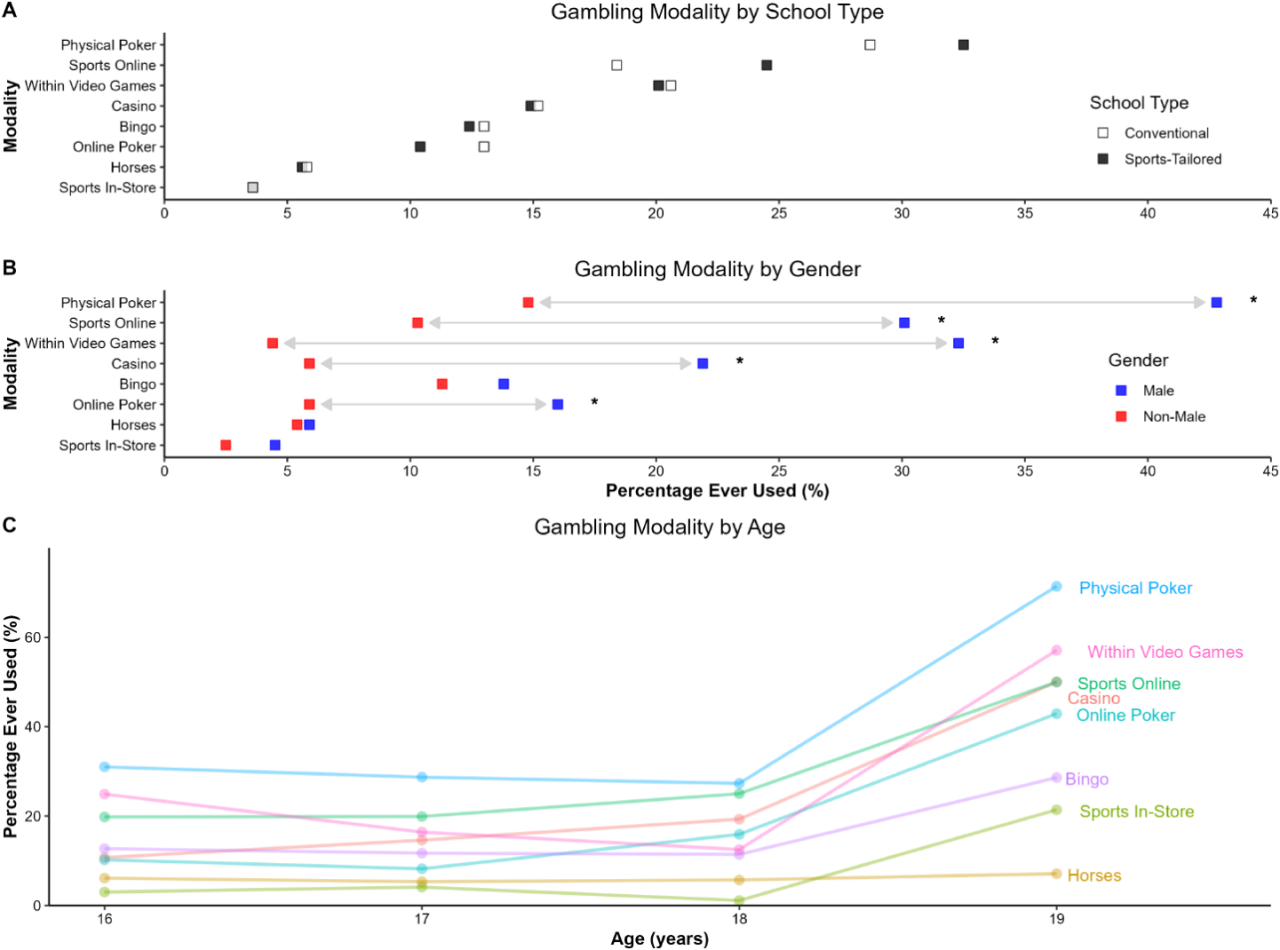
Lifetime Use of Gambling Modalities by School Type, Gender, and Age. Lifetime use of gambling modalities by school type (**A**), gender (**B**), and age (**C**). *p < .05

No significant difference in any gambling type was observed between athletes and non-athletes (*p*s = 0.11-0.99; Fig. 2A). Males were significantly more likely to have gambled on online casino, χ^2^(1) = 23.24, *p* < .001, φ = 0.22, sports betting online, χ^2^(1) = 26.68, *p* < .001, φ = 0.24, online poker, χ^2^(1) = 11.41, *p* < .001, φ = 0.16, and physical poker, χ^2^(1) = 42.53, *p* < .001, φ = 0.30, and gambling within computer games, χ^2^(1) = 55.62, *p* < .001, φ = 0.34, compared with non-males. There were no significant differences between the genders considering the other gambling types, i.e. online bingo, online horse race betting, and land-based sports betting (Fig. 2B). A significant increase of gambling on online casino, χ^2^(1) = 11.72, *r*_*pb*_ = 0.16, *p* < .001 and online poker, χ^2^(1) = 7.03, *r*_*pb*_ = 0.12, *p* = .008, was seen with older age (Fig. 2C).

### Parental Gambling and Participants’ Gambling-Related Attitudes – Participants with or without Problem Gambling

Considering the questions about whether gambling is harmful or beneficial to society, and whether it is morally wrong, a significant difference in attitude was seen between the groups. Problem gambling was positively correlated with the attitudes regarding benefits of gambling, χ^2^(1) = 13.39, *r*_*pb*_ = 0.17, *p* < .001. There was an association between problem gambling and perception of gambling morality, χ^2^(2) = 10.61, *p* = .005, *V* = 0.15. Those who reported problem gambling were more likely to report that gambling was not morally wrong (adjusted standardized residual = 3.2), and less likely to report that they did not know whether it was moral or not (adjusted standardized residual = -2.8). The view on gambling legislation and availability did not differ between those with problem gambling versus those without (*p*s = 0.10–30). There was a significant correlation between problem gambling and ever having felt influenced by gambling advertising to gamble generally, gamble more often or for more money than primarily intended, χ^2^(1) = 72.52, *p* < .001, φ = 0.41. Data prior to binary transformation are presented in Table 1.

**Table 1:** Perceptions of Gambling by Problem Gambling Group

	Problem gambling		
Variable	Yes	No	Total
**Gambling harmful or beneficial to society**			
Harm much greater than the benefits	18 (39%)	271 (64%)	289 (61.2%)
Harm a little greater than the benefits	17 (37%)	95 (22%)	112 (23.7%)
Harm and benefits are equal	4 (8.5%)	43 (10%)	47 (10%)
Benefits a little greater than the harm	4 (8.5%)	11 (2.5%)	15 (3.2%)
Benefits much greater than the harm	3 (7%)	6 (1.5%)	9 (1.9%)
**Morally wrong**			
Yes	6 (13%)	72 (16.9%)	78 (16.5%)
No	30 (65.3%)	179 (42%)	209 (44.5%)
Do not know	10 (21.7%)	175 (41.1%)	185 (39%)
**Legality**			
All types should be legal	14 (30.4%)	81 (19%)	95 (20.1%)
Some types should be legal	28 (60.9%)	318 (74.7%)	346 (73.3%)
All types should be illegal	4 (8.7%)	27 (6.3%)	31 (6.6%)
**Availability of gambling**			
Too available	23 (50%)	208 (48.8%)	231 (48.9%)
Reasonably available	19 (41.3%)	201 (47.2%)	220 (46.6%)
Should be more available	4 (8.7%)	17 (4%)	21 (4.5%)
**Ever affected by gambling advertisement**			
Yes, many times	5 (10.9%)	0 (0%)	5 (1.1%)
Yes, sometimes	13 (28.3%)	16 (3.8%)	29 (6.1%)
No	26 (56.5%)	376 (88.3%)	402 (85.2%)
Never seen any commercial	1 (2.2%)	23 (5.4%)	24 (5.1%)
Do not know	1 (2.2%)	11 (2.6%)	12 (2.5%)

*Note* Which answer best fits your thoughts on whether gambling for money is beneficial or harmful to the society? *p* < .001. Do you think gambling for money is morally wrong? *p* = .005. Which answer fits your thoughts best on whether gambling for money should be legal or not? *p* = .10. What do you think about the availability of gambling for money in Sweden? *p* = .30. Have you ever been affected by gambling advertisement to gamble in general, gamble more often or gamble for more money than first intended? *p* < .001

Similarly, problem gambling was associated with past year paternal gambling, χ^2^(1) = 9.13, *p* = .003, φ = 0.14. Those identified as problem gamblers were more likely to report paternal gambling (50.0%) versus those non-problem gamblers (28.4%). Problem gambling was also associated with maternal gambling, χ^2^(1) = 10.65, *p* = .001, φ = 0.15. Problem gamblers were more likely (28.2%) to report maternal gambling than non-gamblers (11.3%). Additionally, problem gambling was associated with having gambled together with their father (39.1% vs. 13.4%), χ^2^(1) = 20.60, *p* < .001, φ = 0.21, as well as their mother (15.2% vs. 5.9%), χ^2^(1) = 4.36, *p* = .04, φ = 0.11 (Table 2).

**Table 2:** Relationship between child problem gambling and parental gambling

		Yes	No	Total
**Have your father/mother gambled for money during the past 12 months?** **Problem Gambling**				
Father	Yes, often	8 (17.4%)	21 (4.9%)	29 (6.1%)
	Yes, sometimes	15 (32.6%)	100 (23.5%)	115 (24.4%)
	No	21 (45.7%)	247 (58%)	268 (56.8%)
	Do not know	2 (4.3%)	58 (13.6%)	60 (12.7%)
Mother	Yes, often	3 (6.5%)	2 (0.5%)	5 (1.1%)
	Yes, sometimes	10 (21.7%)	46 (10.8%)	56 (11.9%)
	No	29 (63%)	333 (78.2%)	362 (76.7%)
	Do not know	4 (8.7%)	45 (10.6%)	49 (10.3%)
**Have you gambled for money together with your father/mother during the past 12 months?**				
Father	Yes, often	5 (11%)	3 (0.7%)	8 (1.7%)
	Yes, sometimes	13 (28%)	54 (12.7%)	67 (14.2%)
	No	28 (61%)	357 (83.8%)	385 (81.6%)
	Do not know	0 (0%)	12 (2.8%)	12 (2.5%)
Mother	Yes, often	3 (6.5%)	0 (0%)	3 (0.7%)
	Yes, sometimes	4 (8%)	25 (6%)	29 (6.1%)
	No	39 (84.5%)	392 (92%)	431 (91.3%)
	Do not know	0 (0%)	9 (2%)	9 (1.9%)

*Note* Question 1: Have your father/mother gambled for money during the past 12 months? Father *p* = .001, Mother *p* < .001 Question 2: Have you gambled for money together with your father/mother during the past 12 months? Father *p* < .001 Mother *p* < .001

### Parental Gambling and Participants’ Gambling-Related Attitudes – Athlete and Non-Athlete Students

The athlete group had, to a higher extent, a positive view on the impact of gambling on the society compared with the non-athlete group, χ^2^(1) = 9.27, *p* = .002. The moral and legislation aspects of gambling did not differ significantly with respect to whether the participant attended a sports class or not (*p*s = 0.06–63). However, there was a relationship between school type and views on the availability of gambling, χ^2^(2) = 15.89, *p* < .001, *V* = 0.18. A significantly larger number of the participants in sports classes preferred gambling to be more available compared to those in non-sports classes (adjusted standardized residual = 3.1), and significantly fewer participants enrolled in sports classes believed that gambling was too available (adjusted standardized residual = -3.1). No difference was seen between athletes and non-athletes, regarding whether one had felt influenced to gamble in general or to gamble for more money than intended after seeing a gambling advertisement, χ^2^(1) = 0.14, *p* = .70 (Supplementary Table 1).

Participants attending a sports class were more likely to report that their fathers had gambled in the past year, χ^2^(1) = 9.06, *p* = .003, φ = 0.14, and that they had gambled with their father, χ^2^(1) = 5.66, *p* = .02, φ = 0.11. School curriculum was not associated with past year maternal gambling, χ^2^(1) = 2.56, *p* = .11, nor gambling alongside their mother, χ^2^(1) = 0.17, *p* = .68 (Supplementary Table 2).

### Psychological Distress

Psychological distress was not associated with problem gambling. No difference in scoring above cut-off for psychological distress was seen in respondents with a likely problem gambling compared with those without a likely problem gambling, χ^2^(1) = 0.16, *p* = .69 (Supplementary Table 3).

## Discussion

### Gambling Problems and Correlating Factors

The present study investigated a sample of upper-secondary school students´ gambling habits, their relationship with gambling and their own assessment of their mental health. Based on previous literature in the area, it was also examined whether there were any differences between students enrolled in sports-oriented classes and conventional classes.

In the present study, almost 10% of the 472 participants endorsed at least one of the NODS-CLiP-questions, indicating lifetime problem gambling. This outcome is in line with previous data from The Swedish Public Health Agency, which showed that in 2023, 10% of boys and just under 1% of girls in school year 11 engaged in risky gambling (Public Health Agency of Sweden, 2023). Similarly, there was a higher incidence of gambling problems in males compared with females, 13.4% of the males in this study endorsed problem gambling, compared to 4.9% of the non-males. The fact that more male than female upper secondary school students exhibit a risky gambling behavior is also in agreement with prior observations, where, among young individuals, problem gambling is markedly more prevalent in boys than in girls (Claesdotter-Knutsson et al., 2022).

Males tended to have gambled using casino games, sports betting online, both online and physical poker and within online games. Previous studies have shown that males tend to gamble more on strategy-based gambling sites, while females are more prone to participate in less interactive gambling modalities, such as online casinos (Håkansson et al., 2017; Håkansson & Widinghoff, 2020). This was not as clear among these young participants, as males reported higher prevalence of gambling overall, regardless of the gambling type. This merits further investigation, in order to examine whether findings of gender differences typically reported in adults may not hold among adolescents in these age groups.

A positive association between age and problem gambling was observed. Likewise, a significant increase in gambling on casino, sports online and online poker was seen in older age groups. Many teenagers gain more freedoms with age, such as unlimited internet-time and access to personal assets, making them more exposed to the risk of developing problem gambling. The increase between the age of 17 and 18 can also be explained by the fact that the legal age of gambling in Sweden is 18. At 18, one is also considered an adult and parents do not necessarily exert as much control over their children´s finances or behavior. An exception of this pattern was seen between the age of 16 and 17, where the prevalence instead appeared to decrease. This could be due to various factors, for example, unequal number of participants in the age groups or the sample size being too small for differences to be observed.

There was a significant correlation between endorsing problem gambling and being influenced by gambling advertising. Having been exposed and affected by a gambling advertisement could both result in gambling in general, gambling more often or gambling for a larger amount of money than firstly intended. The correlation between exposure to gambling advertising and a higher level of gambling problem has been described in a previous Norwegian study which also showed that internet advertising had the strongest influence (Syvertsen et al., 2022).

Considering the perceptions that participants had on gambling’s societal impact, differences were observed between those identified as problem gamblers versus those that did not meet the threshold for problem gambling. Individuals who reported problem gambling tended to choose the alternatives where the perceived benefits exceeded the harm. In the problem gambling group, 65% did not think of gambling as morally wrong, while only 42% in the non-problem gambling-group did. Participants’ opinions on the legal status of gambling also differed, with problem gamblers tending to have a more liberal view on gambling legislation, though this result was not statistically significant. Based on these results, it is not possible to determine whether having a more positive view on gambling predisposes students to problem gambling or if people generally tend to have more positive perceptions of the activities they are personally involved in. Here, more research is needed in community and clinical settings.

It seemed likely that the gambling habits of both parents were associated with the gambling habits of their child. More frequent gambling among the parents correlated significantly with a tendency to gamble among the participants. Also, gambling together with any of the parents showed an association with an increased risk of problem gambling. Just like other risk behaviors, such as alcohol use, the gambling habits of the parents have shown to affect the child´s risk of developing a similar issue (Vachon et al., 2004).

### Gambling among Young Athletes

The present study was carried out in light of previous findings of an association between being active in different types of sports and having an increased risk of gambling problems (Vachon et al., 2004). However, this study did not show any significant differences between athletes and non-athletes regarding the risk of endorsing problem gambling behavior. Also, no significant differences in any gambling types were observed between athletes and non-athletes. The largest difference – in absolute numbers and percentages – was seen in gambling on sports online, although the difference was modest. This could be because athletes, in general, have a greater interest in sports and are more exposed to betting in that context. At the same time, the prevalence of a likely gambling problem and the prevalence of gambling on each gambling type did not differ between those reporting practicing a team’s sport and those participating in an individual sport.

Altogether, the disparities found between athletes and non-athletes in the literature, primarily focusing on elite athletes, were generally not demonstrated in this study in high school students. Also, the present study did not demonstrate any difference between team sports and other sports, and this lack of difference was consistent with previous findings among young athletes in the present setting (Håkansson et al., 2018). In a review of previous research, one study in elite athletes did not demonstrate a risk increase in comparison to the general population, whereas the remaining studies did, making the present findings somewhat surprising (Håkansson et al., 2021).

One explanation of the lack of differences seen here may be that being a student athlete at the high school level, as was the case in the present study, may not involve the same level of sports achievements as in previous studies in elite athletes. Although head-to-head comparisons are few between student-athletes and non-athletes high school students, the present findings are in contrast to some previous reports of an increased risk in student-athletes in the US (Weiss, 2010). In that research, a risk increase was seen for male student-athletes but not for their female counterparts (Weiss, 2010), which is thereby in line with elite athlete research describing an increased prevalence in male, but not female, athletes (Vachon et al., 2004). Altogether, the prevalence and correlates of problem gambling in high school students, with or without sports involvement, merits further investigation in the present geographical setting, and elsewhere.

However, while the prevalence of problem gambling and gambling experience did not differ between athletes and non-athletes, some differences were instead seen for gambling-related attitudes. More participants in sports-classes had a positive view on the effects of gambling on the society compared with participants in non-sports classes. A possible explanation to this outcome is the relationship athletes have with gambling through their involvement in sports. For example, gambling operators are common sponsors of sports activities and typically seen in advertising related to sports events (Deans et al., 2017). Today, gambling is seamlessly integrated into sporting events, as virtually all professional sports are available for betting.

Student-athletes in our sample tended to think that gambling should be more available than it already is. This distribution is interesting and can possibly be explained by the restrictions on sports betting. Betting together with a parent or having a mother who gambled more frequently were not more common in any of the groups. Having a father who frequently gambled was more typical among the athletes.

Thus, although differences in actual gambling patterns and problems were few or non-existing, the present study did confirm differences in gambling-related attitudes between athletes and non-athletes. Previous research, including qualitative work with athletes and other professions within the world of elite sports, has suggested that attitudes towards gambling in sports favor the development of problem gambling (Vinberg et al., 2021), also in combination with the substantial component of gambling in sports-related advertising (Deans et al., 2017). Thus, although a risk increase in problem gambling could not be demonstrated here, the gambling-related attitudes described here can be considered to favor the pattern seen in previous research in more established elite athletes (Håkansson et al., 2021), where significant gambling problems have developed. This also lends support to future preventive work or interventions targeting young individuals, in order to prevent favorable gambling attitudes to cause larger gambling problems subsequently. In the present setting, some degree of preventive work in elementary schools and high schools is carried out in Sweden, but has involved primarily prevention of tobacco, alcohol and drug use, and only recently started to involve gambling. Thus, gambling prevention oriented towards adolescents can be assumed to be limited and may have potential for improvement. Also, to the best of the authors’ knowledge, it is unlikely that sports-oriented high schools provide more extensive gambling-preventive intervention than other schools. However, given the absence of differences between different schools included here, further preventive efforts may be beneficial in all types of high school environment, the present study paradoxically, despite not showing increased gambling in sports high schools, still lends support to the targeting of athlete environments regarding attitudes towards gambling.

### Limitations

This study involved upper secondary school students from the Skåne region, Sweden and was conducted within a limited time span. This may lead to the results not being representative on a national or an international population. Therefore, if a population from another area participated in the same study, the results could have been different compared to the ones presented here.

Just like other measure tools, both NODS-CLiP and the Kessler-6 scale have limitations. There are aspects that the forms do not address, which could result in loss of certain valuable information. The NODS-CliP questionnaire is consisting of only three questions, with the purpose of being a quick screening tool. At the same time, gambling problem is a complex issue, requiring further investigation to be properly assessed. The Kessler-6 scale is also addressing only the past six months, which may not give the right insight in the participant´s actual mental health.

More males than non-males participated in the survey, which could have had an impact on the results. At sports schools, 68% of respondents were boys, and at non-sports schools, their proportion was 48%. Thus, the gender distribution in non-sports schools is very close to that of the general population. For sports high school students participating in the present study, the male predominance is slightly larger than in sports high schools in general in Sweden; in a recent national survey study of sports high schools in Sweden, 58% of respondents were male (Andersson et al., 2023). For example, the number of males vs. females attending a sports-oriented class could have biased the outcome, but if so, this would rather increase the rates of gambling problems among respondents at sports high schools, given the higher rates of gambling in young men than in women (Abbott et al., 2018; Claesdotter-Knutsson et al., 2022). Another aspect to take in consideration is that the “non-male-group” included females, other gender identities and those who preferred not to answer the question. A better implementation, to obtain a more reliable result, could have been to separate each group, which, however, would not have allowed for statistically robust comparisons across the smaller groups.

## Conclusion

This study in upper secondary students in sports schools and non-sports schools did not show any significant differences between athletes and non-athletes. Yet, it does not change the fact that athletes are more often exposed to gambling via their involvement in sports, and attitudes favoring gambling were more common in athletes than in non-athletes. Therefore, the preventive efforts made within sporting associations are still of great importance. A correlation was seen between exposure to gambling advertising and problem gambling. This highlights the need for authorities to address, for example, the restriction of gambling operators’ possibilities to advertise on websites typically used by many young individuals.

## Electronic Supplementary Material

Below is the link to the electronic supplementary material.

Supplementary Material 1

## Data Availability

Non-identified data can be shared upon relevant request to the authors.
