# Supplementary material for: Gambling Habits and Attitudes among Athlete and Non-Athlete High School Students in Skåne Region, Sweden
Source: J Gambl Stud. 2024 Jul 12;41(1):203–17. doi: 10.1007/s10899-024-10333-3 (PMC11861237; doi:10.1007/s10899-024-10333-3)
Supplement: Supplementary file 1 — Supplementary Material 1 [file 10899_2024_10333_MOESM1_ESM.docx]

**Supplementary Table 1. Gambling attitudes, comparison of sports and non-sports classes. Pearson Chi-Square.**

| **Harmful or Beneficial to the society** | **Sports class** | | | | | | | | |  |  |  |  |
| --- | --- | --- | --- | --- | --- | --- | --- | --- | --- | --- | --- | --- | --- |
|  | Yes | | | No | | | Total | | |  |  |  |  |
| The harm of gambling is much greater than the benefits | | | 137 (55.1%) | | | 152 (68.2%) | | | 289 (61.2%) | | |  |  |
| The harm of gambling is a little greater than the benefits | | 67 (26.9%) | | | 45 (20.2%) | | | 112 (23.7%) | | |  |  |  |
| The harm and benefits of gambling are equal | | 27 (10.8%) | | | 20 (9%) | | | 47 (10%) | | |  |  |  |
| The benefit of gambling is a little greater than the harm | | 11 (4.4%) | | | 4 (1.8%) | | | 15 (3.2%) | | |  |  |  |
| The benefit of gambling is much greater than the harm | | 7 (2.8%) | | | 2 (0.8%) | | | 9 (1.9%) | | |  |  |  |
| **Morally wrong** |  | | | | | | | | |  |  |  |  |
|  |  | | |  | | |  | | |  |  |  |  |
| Yes | 38 (15.3%) | | | 40 (17.9%) | | | 78 (16.5%) | | |  |  |  |  |
| No | 123 (49.4%) | | | 86 (38.6%) | | | 209 (44.3%) | | |  |  |  |  |
| Don´t know | 88 (35.3%) | | | 97 (43.5%) | | | 185 (39.2%) | | |  |  |  |  |
| **Legislation** |  | | | | | | | | |  |  |  |  |
|  |  | | |  | | |  | | |  |  |  |  |
| All types should be legal | 54 (21.7%) | | | 41 (18.4%) | | | 95 (20.1%) | | |  |  |  |  |
| Some types should be legal | 178 (71.5%) | | | 168 (75.3%) | | | 346 (73.3%) | | |  |  |  |  |
| All types should be illegal | 17 (6.8%) | | | 14 (6.3%) | | | 31 (6.6%) | | |  |  |  |  |
| **Availability of gambling** | | |  | | | | | | | | |  |  |
|  |  |  |  | | |  | | |  | | |  |  |
| Gambling is too available | | | | | 105 (42.2%) | | | 126 (56.6%) | | | 231 (48.9%) | | |
| Gambling accessibility is reasonable as it is | | | | | 126 (50.6%) | | | 94 (42%) | | | 220 (46.6%) | | |
| Gambling should be more available | | | | | 18 (7.2%) | | | 3 (1.4%) | | | 21 (4.5%) | | |
| **Ever been affected by gambling advertisement** | |  | | | | | | | | |  |  |  |
|  |  |  | | |  | | |  | | |  |  |  |
| Yes, many times | | 3 (1.2%) | | | 2 (0.9%) | | | 5 (1.1%) | | |  |  |  |
| Yes, sometimes | | 16 (6.4%) | | | 13 (5.8%) | | | 29 (6.1%) | | |  |  |  |
| No | | 215 (86.4%) | | | 187 (83.9%) | | | 402 (85.2%) | | |  |  |  |
| Never seen any commercial | | 8 (3.2%) | | | 16 (7.2%) | | | 24 (5.1%) | | |  |  |  |
| Don´t know | | 7 (2.8%) | | | 5 (2.2%) | | | 12 (2.5%) | | |  |  |  |

Question: Which answer best fits your thoughts on whether gambling for money is useful or harmful to the society? P=0.029. Do you think gambling for money is morally wrong? P=0.060. Which answer fits your thoughts best on whether gambling for money should be legal or not? P=0.628. What do you think about the availability of gambling for money in Sweden? P<0.001. Have you ever been affected by gambling advertisement to gamble in general, gamble more often or gamble for more money than firstly intended? P= 0,401

**Supplementary Table 2. Maternal and paternal gambling, comparison of sports and non-sports classes. Pearson Chi-Square.**

|  |  |  |  |  |
| --- | --- | --- | --- | --- |
| **F a t h e r M o t h e r F a t h e r M o t h e r** | **Have your father/mother gambled for money during the past 12 months?**  **Sports Class** | | | |
|  |  | Yes | No | Total |
|  | Yes, often | 22 (8.9%) | 7 (3.1%) | 29 (6.1%) |
|  | Yes, sometimes | 69 (27.8%) | 46 (20.6%) | 115 (24.4%) |
|  | No | 126 (50.4%) | 142 (63.7%) | 268 (56.8%) |
|  | Don´t know | 32 (12.9%) | 28 (12.6%) | 60 (12.7%) |
|  |  |  |  |  |
|  | Yes, often | 1 (0.4%) | 4 (1.8%) | 5 (1%) |
|  | Yes, sometimes | 37 (14.9%) | 19 (8.5%) | 56 (11.9%) |
|  | No | 185 (74.3%) | 177 (79.4%) | 362 (76.7%) |
|  | Don´t know | 26 (10.4%) | 23 (10.3%) | 49 (10.4%) |
|  | Total | 249 | 223 | 472 |
|  | **Have you gambled for money together with your father/mother during the past 12 months?** | | | |
|  | Yes, often | 4 (1.6%) | 4 (1.8%) | 8 (1.7%) |
|  | Yes, sometimes | 45 (18.1%) | 22 (9.9%) | 67 (14.2%) |
|  | No | 193 (77.5%) | 192 (86.1%) | 385 (81.6%) |
|  | Don´t know | 7 (2.8%) | 5 (2.2%) | 12 (2.5%) |
|  |  |  | | |
|  | Yes, often | 1 (0.4%) | 2 (0.9%) | 3 (0.6%) |
|  | Yes, sometimes | 17 (6.8%) | 12 (5.4%) | 29 (6.1%) |
|  | No | 226 (90.8%) | 205 (91.9%) | 431 (91.4%) |
|  | Don´t know | 5 (2%) | 4 (1.8%) | 9 (1.9%) |
|  | Total | 249 | 223 | 472 |

*Question 1: Have your father/mother gambled for money during the past 12 months? Father P=0.007 Mother P=0.088*

*Question 2: Have you gambled for money together with your father/mother during the past 12 months? Father P=0.078 Mother P=0.825*

**Supplementary Table 3. Psychological distress in individuals with and without problem gambling.**

| **Psychological distress (at least 5 points on Kessler-6 score)** | **Problem gambling** | | |
| --- | --- | --- | --- |
|  | Yes | No | Total |
| Yes | 32 (69.6%) | 293 (68.8%) | 325 (68.9%) |
| No | 12 (26.1%) | 123 (28.9%) | 135 (28.6%) |
| Excluded*** | 2 (4.3%) | 10 (2.3%) | 12 (2.5%) |
| Total | 46 | 426 | 472 |

Chi-square Linear-by-linear, p=0.680. *Exclusion due to one or more missing items, could not be determined whether the total score of the individual summed up to 5 points or not.
